# Functional Connections Between Insulin and Neurotrophin-3 in Hemodialysis Patients with Type 2 Diabetes

**DOI:** 10.3390/cimb48090884

**Published:** 2026-08-30

**Authors:** Mihaela Gheorghiu, Maria-Florina Trandafir

**Affiliations:** Pathophysiology and Immunology Department, “Carol Davila” University of Medicine and Pharmacy, 050474 Bucharest, Romania; maria.trandafir@drd.umfcd.ro

**Keywords:** end-stage renal disease, diabetes mellitus, insulin, Neurotrophin-3

## Abstract

The bidirectional transfer of biological information between insulin and neurotrophin-3 (NT-3) in hemodialysis patients (end-stage renal disease—ESRD) with type 2 diabetes mellitus (T2DM) is extremely poorly studied. Why was NT-3 chosen? Because, of all the nerve growth factors, this one also exhibits specific actions at the nephron level. The authors of this manuscript also conducted the first study on the serum behavior of NT-3 in this category of patients. Even though the results have already been published, the way in which an endocrine compound (insulin) interacts at the renal level with a growth factor with predominantly autocrine and paracrine action (NT-3), under both normal and pathological conditions, has not been thoroughly studied. This paper will address the following topics: 1. that insulin synthesis and release involve the same molecules that also exert important actions at the renal level; 2. involvement of insulin deficiency/resistance in the development of diabetic nephropathy and end-stage renal disease vs. kidney influences on serum glucose and insulin levels; 3. actions of NT-3 under normal conditions vs. diabetes mellitus; 4. aspects highlighted by personal studies performed on hemodialysis patients with T2DM. Taking an original approach, the functional link between insulin and NT-3 in hemodialysis patients, especially those with T2DM, will be presented. Finally, new therapeutic possibilities and directions for patient monitoring will be proposed, as well as topics for further research.

## 1. Introduction

Type 2 diabetes mellitus (T2DM) is the most common form of diabetes, affecting over 828 million people worldwide and representing over 90% of all diabetes cases [1]. It presents a rising trend, the number of cases having more than doubled in the last two decades, with projections reaching 853 million by 2050 [1]. On the other hand, 2023 global statistics indicated roughly 850 million cases of chronic kidney disease [2], of which more than 2.7 million require hemodialysis, a number expected to reach 5.4 million in 2030 [3]. This parallel presentation of the two pathologies’ prevalence is not accidental: chronic kidney disease (CKD) and end-stage renal disease (ESRD) are frequent complications of diabetes [4].

This article aims to present the functional connections between insulin and neurotrophin-3 (NT-3) in diabetic hemodialysis patients. This is a novel approach, as the authors of this review also conducted the first study on NT-3 in this category of patients. Although this research was conducted on a limited number of patients, it is statistically significant for hemodialysis patients in Bucharest, Romania. Therefore, the results obtained should be both popularized and further studied to reach a deeper level of understanding of the mechanisms underlying them [5,6,7]. Also, hemodialysis patients, including diabetic patients, are less studied due to the minimal chances of improving the two associated pathologies and their consequences. However, it is known that many pathophysiological mechanisms are only highlighted in borderline situations similar to the two diseases, and an additional, more in-depth study could discover, at least theoretically, therapeutic approaches that the researcher would not think of under normal conditions.

Another aspect that may stimulate the reader’s curiosity is the presentation of the exchange of biological information between two different levels of the reality that constitutes the human organism: the level of insulin, which normally has serum concentrations on the order of 0.5–2 ng/mL [8], and the level of NT-3, which some studies estimate at 5–20 pg/mL, approximately 100 times lower than insulin [9].

The third interesting aspect is represented by the unique functional characteristics, including those at the renal level, of NT-3, which sets it apart from nerve growth factor (NGF) and brain-derived neurotrophic factor (BDNF).

The fact that this article addresses two associated pathologies (diabetes and ESRD) in the same individual forces the authors to present the effects that tissue insulin deficiency/resistance has on renal function. However, an approach only in the direction of diabetes → ESRD is not sufficient, since it has long been known that the kidney has a major influence on serum glucose and insulin levels [10,11]. This bidirectional approach will also address the functional connections between insulin and NT-3, starting from receptors and intracellular signaling mechanisms, which have many common features. Since NT-3 has not been studied in patients with T2DM and ESRD, nor in those with ESRD alone, only the authors’ observations from the previously performed quantitative study will be presented. However, it is important to note that this paper is a review and not an article that presents in detail the quantitative results of already published determinations.

These are the reasons why the following topics will be addressed in this manuscript.

## 2. Relevant Studies

### 2.1. The Synthesis and Release of Insulin Involve the Same Molecules That Exert Important Actions at the Renal Level

**Scientific importance of the topic:** *The reader is informed about the functional interconnections between insulin and the molecules that influence its synthesis and release. Behind each of these molecules is the organ that synthesizes it and the biological “interest” of this organ in the synthesis and release of insulin. The reader learns that all of these molecules also exert important actions at the renal level.*

Although officially considered to have been discovered in 1921 by Banting and Best, insulin was identified and isolated as early as 1914 from the canine pancreas by the Romanian scientist Nicolae Paulescu, initially receiving the name pancrein [12].

It is a peptide hormone consisting of 51 amino acids that organize as a hexamer consisting of three heterodimers [13]. Insulin is synthesized as a 110-amino acid preprohormone, which has a signal peptide at its N-terminus that guides insulin transport into the RER (rough endoplasmic reticulum) and which is then cleaved by peptidases in the Golgi reticulum [14]. The result is proinsulin, which is hydrolyzed by proconvertases 1, 2 and 3 and by carboxypeptidase, with the formation of two chains of insulin and C-peptide, in an equimolar mixture [15].

Insulin is synthesized, stored in secretory granules and released into the blood by pancreatic beta cells in the islets of Langerhans. The major stimuli for insulin secretion are: serum glucose level (must be at least 80–100 mg/dL), acetylcholine, melatonin, estrogens, oxytocin, leptin, human growth hormone (GH/STH) and incretins (GLP-1—Glucagon-Like Peptide-1 and GIP—Glucose-Dependent Insulinotropic Polypeptide) [14,16].

It is useful to very briefly present the functional interrelationships between insulin and the hormones that influence its release.

**Acetylcholine (ACh)** intensely **stimulates insulin production** from pancreatic beta cells during postprandial periods. It is the prototypical parasympathetic hormone released massively at the level of the pancreatic endings of the vagal nerve. Pancreatic beta cells bind ACh to specific muscarinic receptors (M3Rs) belonging to the GPCR (G protein-coupled receptor) family [17]. The result is the submembrane activation of PLC (phospholipase C) and the initiation of the PIP2 (phosphatidylinositol biphosphate) cascade, with the production of IP3 (inositol triphosphate) and DAG (diacylglycerol). These molecules signal through separate but convergent pathways involving Ca^2+^ and ultimately generate the activation of cytoskeletal proteins and the mobilization of insulin granules to the outer membrane and their extracellular release. It is very interesting to note that **renal cells, especially podocytes, synthesize and secrete ACh**, **which has an anti-inflammatory role, acting as an endogenous protective mechanism at the level of glomerular endothelial cells.** Locally produced ACh is added to that released from renal nerves [18].

**Melatonin** is synthesized during the night in the pineal gland, from where it is released into the blood [19]. Its synthesis is inhibited during the day. Melatonin is an indoleamine that binds to specific membrane receptors MT1R and MT2R, belonging to the GPCR family. The number of MT2Rs is several times higher than that of MT1Rs in both alpha and beta pancreatic cells. In contrast, there is no difference in the number of MT2Rs between the two cell types [20]. The two types of receptors can be found on the membrane as distinct monomers, or they can associate in the form of a heterodimer. Only as a heterodimer can they transmit activating signals from melatonin to the cell to which they belong (PLC activation, with IP3 production) [19]. When melatonin binds to MT1R or MT2R in monomeric form, transduction via the adenylate cyclase (AC) pathway is activated, which ultimately results in the inhibition of insulin release [21]. **These different modes of signal transduction generated by melatonin represent a receptor-mediated autoregulation mechanism of insulin discharges from beta cells**. It can be concluded that low melatonin levels increase the risk of diabetes. It is very important to remember that, in addition to the antidiabetic effects of increased concentrations of melatonin, **it also has essential actions at the renal level.** Thus, it has an **antioxidant action**, directly neutralizing free radicals (ROS) and stimulating the antioxidant defenses of the body, including the kidney [22,23]. It also has **anti-inflammatory effects**, by decreasing the release of pro-inflammatory cytokines in the kidney [22,23]. Melatonin also exerts an **antiapoptotic role** through antioxidant protection of mitochondria in kidney cells, thus aiding normal cellular ATP production [22,23]. A very important role is also played by its **antifibrotic properties**, as melatonin inhibits the action of fibroblasts in the kidneys [22,23]. This action is deficient in CKD, fibrosis being a defining characteristic of this pathology.

**Estrogens stimulate insulin release** from pancreatic beta cells, and **inhibit their apoptosis** by binding to the membrane estrogen receptors [24]. Through these actions, they improve tissue tolerance to glucose. These estrogen effects explain why premenopausal women have a lower risk of type 2 diabetes compared to men or postmenopausal women. It has been shown that the **pancreas can synthesize estrogen metabolites,** such as catechol estrogens [25]. This indicates the action of estrogens at the pancreatic level, although there is no clear evidence of their synthesis in this organ. Another proven action of estrogens, closely related to the subject of this article, is **kidney protection against ferroptosis and acute kidney injury** through both nongenomic and genomic mechanisms [26].

**Oxytocin**: Although it is known that the major synthesis of oxytocin takes place in the hypothalamus, synthesis is currently also proven in the heart, ovaries, uterus, and placenta, adrenal medulla, and **pancreas,** and even parts of the brainstem and spinal cord [27]. Another very important piece of information is the **evidence of renal synthesis of oxytocin**. At this level, oxytocin stimulates natriuresis by binding to the V2R receptors of vasopressin (antidiuretic hormone) and stimulating an increase in the number of aquaporins (AQPs) 2 and 3 at the level of the collecting tubules [28]. Another action, convergent with those presented above, is the stimulation of renin secretion, by activating both its own receptors (OTR) and beta-adrenergic receptors [29]. Aldosterone, which is the final product of this pathway, acts synergistically with oxytocin.

It is known that insulin is produced by pancreatic beta cells, but it has recently been shown that alpha cells also participate in this phenomenon. They secrete “intra-inslet” GLP-1, and **oxytocin stimulates the synthesis and release of GLP-1 from alpha cells**. In turn, GLP-1 stimulates beta cells to produce and release insulin when blood glucose levels are high [30].

**Leptin** is a hormone secreted by adipocytes that has a key role in regulating glucose homeostasis, acting through both central and peripheral mechanisms. At the central level, leptin signals satiety to the brain, reducing appetite and decreasing glucose-stimulated insulin secretion [31]. In the periphery, unlike the hormones presented so far, **leptin inhibits insulin release, while insulin stimulates leptin release**. In addition to the adipose tissue, it is also secreted in lymphoid tissues, the placenta, and ovaries [31]. The leptin receptor on the beta cell membrane belongs to the class I cytokine receptor family [32]. The main signaling pathway initiated involves the activation of JAK (Janus kinase) and STAT (Signal Transducers and Activators of Transcription) transcription factors [31,33]. Unlike all the hormones listed above, leptin is not also produced in the kidneys. But the kidneys play a crucial role in removing leptin from the bloodstream by glomerular filtration and degradation. Therefore, **serum leptin levels are significantly increased in CKD and worsen diabetes by inhibiting insulin secretion** [34].

**Growth hormone (GH) or somatotropin hormone (STH)** exerts a dual effect on glucose metabolism: on the one hand, **it stimulates pancreatic beta cells to release insulin, and on the other hand, it increases peripheral insulin resistance** [35]. Thus, under the action of GH, secretory pancreatic beta cells synthesize and secrete both insulin and growth hormone-releasing factors (GHRFs), such as HPGRF (Human Pancreatic Growth Hormone-Releasing Factor) [36]. It is important to note, however, **that pancreatic cells also produce and release GH, stimulating their own insulin secretion by autocrine and paracrine mechanisms.** Certain pancreatic tumors can synthesize GH in excess [35]. GH induces insulin resistance in insulin-dependent tissues (liver, muscle, adipose tissue). Thus, in the liver it stimulates gluconeogenesis and reduces the ability of insulin to suppress it. In muscle and adipose tissues, it decreases the absorption and peripheral utilization of glucose, stimulating lipolysis [35,36]. In these tissues GH behaves as an insulin antagonist hormone, causing the body to need more insulin to manage blood sugar levels.

The **kidneys are major targets for GH**, with podocytes and tubular cells expressing GH receptors and producing the primary mediator of GH, IGF-1 (insulin-like growth factor-1). Thus, GH contributes to the regulation of kidney function, cell growth, and electrolyte balance, although excess GH can harm the kidneys in disease states, especially diabetes [37,38].

**Incretins (GLP-1 and GIP)** are polypeptides secreted by the intestine after food ingestion that **stimulate insulin secretion** from pancreatic beta cells in a glucose-dependent manner. GLP-1 is produced by L-cells in the lower gut (ileum and colon). In addition to stimulating insulin secretion from pancreatic beta cells (by binding to specific membrane receptors of the GPCR family), it decreases glucagon release, slows gastric emptying, and decreases appetite. Through these actions, GLP-1 is particularly effective in type 2 diabetes [39]. GIP is secreted by K cells in the upper small intestine (duodenum/jejunum), stimulated by ingested food. It exerts the same actions as GLP-1, but has a lesser impact on glucagon release [39].

Incretins, especially GLP-1, **exert important protective actions at the renal level**: they reduce inflammation and its secondary fibrosis, produce vasodilation of glomerular afferent arterioles, protect glomerular structures (reduce podocyte damage and inhibit glomerular basement membrane thickening and mesangial expansion) and increase natriuresis [40,41]. All these actions contribute to reducing albuminuria and maintaining the glomerular filtration rate (GFR), which is **important for slowing the progression of diabetic kidney disease** (DKD).

The effects of the classical insulin antagonists (catecholamines, glucagon and cortisol) have not been discussed, given that they are well known. It should only be noted that catecholamines (especially adrenaline) and glucagon act directly on pancreatic beta cells by binding to specific membrane receptors. The results are complex, often dual (stimulatory/inhibitory). On the other hand, cortisol stimulates insulin resistance and generates long-term disorders of beta cell function [42].

### 2.2. Involvement of Insulin Deficiency (or Resistance) in the Development of Diabetic Nephropathy and End-Stage Renal Disease vs. Kidney Influences on Serum Glucose and Insulin Levels

**Scientific importance of the topic:** *The reader is informed about the general mechanism of insulin signaling at the cellular level and about its particularities in the nephron (glomeruli and tubules). They also learn about the actions of insulin in the nephron segments. The fundamental idea of this topic is that, although the kidney is not an insulin-dependent tissue, it actively participates in maintaining a normal insulin cycle in the body. Finally, the reader is informed about the mechanisms by which insulin resistance induces CKD.*

**Figure 1:**
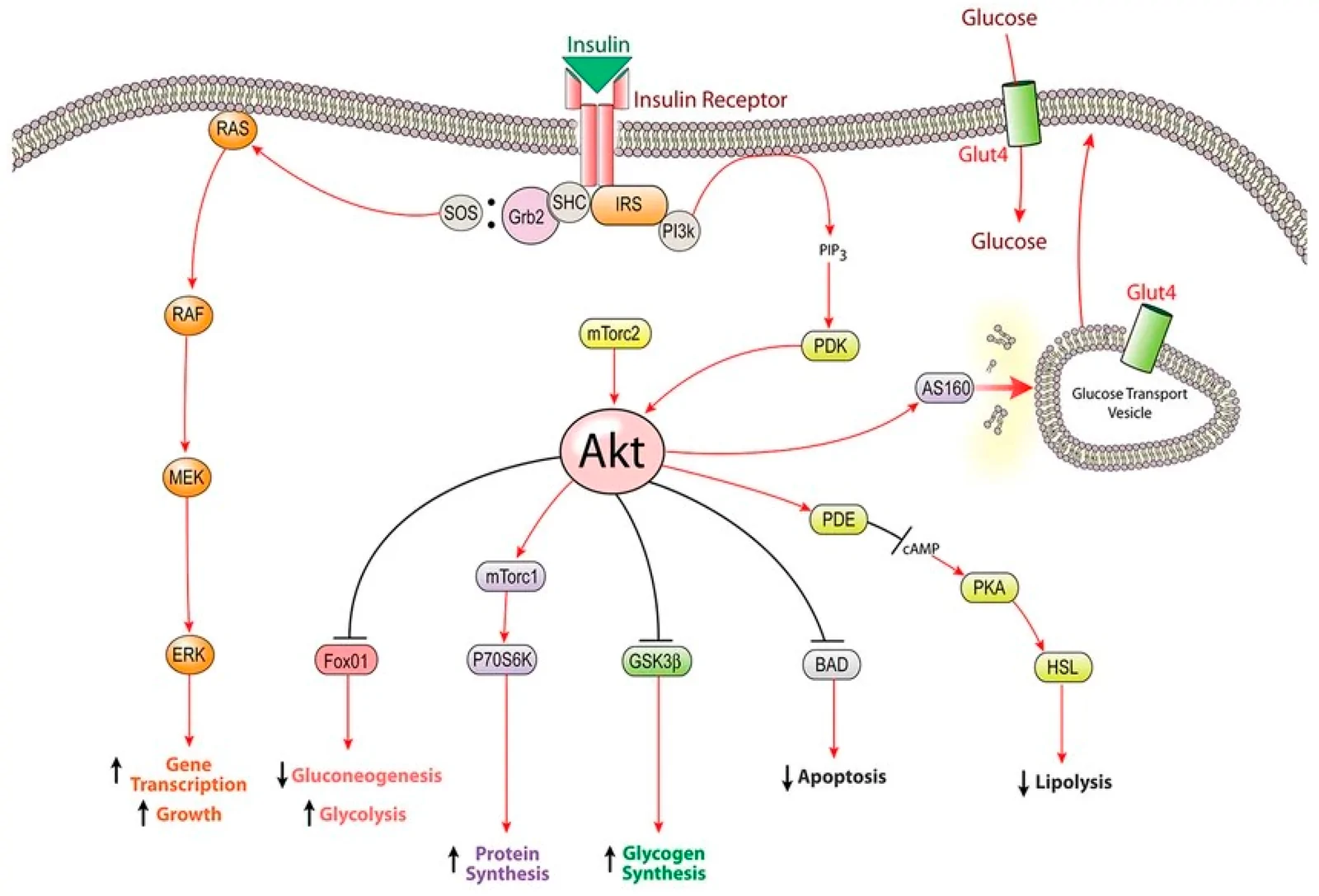
Insulin signaling cascade: insulin binds to tyrosine kinase receptors, leading to the activation of both the PI3K-Akt pathway and the MAPK pathway. Abbreviations used: insulin receptor substrate (IRS), glycogen synthetase kinase β (GSK3β), extracellular signal-related kinase (ERK), Srchomology-2-containing protein (Shc), forkhead box protein O1 (FOXO1), Akt substrate of 160 kDa (AS160), mammalian target of rapamycin complex (mTorc), p70 ribosomal protein S6 kinase (p70S6K), hormone-sensitive lipase (HSL), phosphodiesterase (PDE), protein kinase A (PKA), phosphoinositide-dependent kinase (PDK), growth factor receptor-bound protein 2 (Grb2), son of sevenless (SOS), glucose transporter 4 (Glut4). Reprinted from ref. [46].

The insulin receptor (InsR) is a peptide heterodimer consisting of transmembrane subunits α and β. Functionally, it belongs to the family of growth factor receptors (type II), which are tyrosine kinase receptors (RTKs) [43]. They frequently interact with GPCRs, another receptor family used by hormones [44]. After insulin binding, transreceptor signaling begins through conformational changes in the β subunit, resulting in phosphorylation of the enzymatic site and activation of tyrosine kinase functions [45]. After signal uptake by several adaptor proteins (SHC, IRS, Grb2), the next key points in the signaling pathway are the activation of PI3K (phosphatidylinositol 3-kinase) and then of Akt/PKB (protein kinase B) kinase (Figure 1) [45,46].

Through all these signaling mechanisms, glucose homeostasis is ensured, as well as the general anabolic effects of insulin: the biosynthesis of proteins, lipids and glycogen.

**These receptors are also present at the renal level, on all cell types (podocytes, mesangial cells, tubular nephrocytes)** [47], so through them insulin exerts many effects. Being a small molecule, insulin is completely filtered through the glomeruli and must be reabsorbed in the proximal convoluted tubule. This behavior explains the high concentrations of insulin that both podocytes and proximal nephrocytes come into contact with.

**At the glomerular level,** podocytes are the main cell type. Under normal conditions, they emit finger-like extensions around the basement membrane of the glomerular capillaries [15]. Between these cells there are junctions that allow the appearance of very small spaces through which glomerular filtration is achieved.

**Figure 2:**
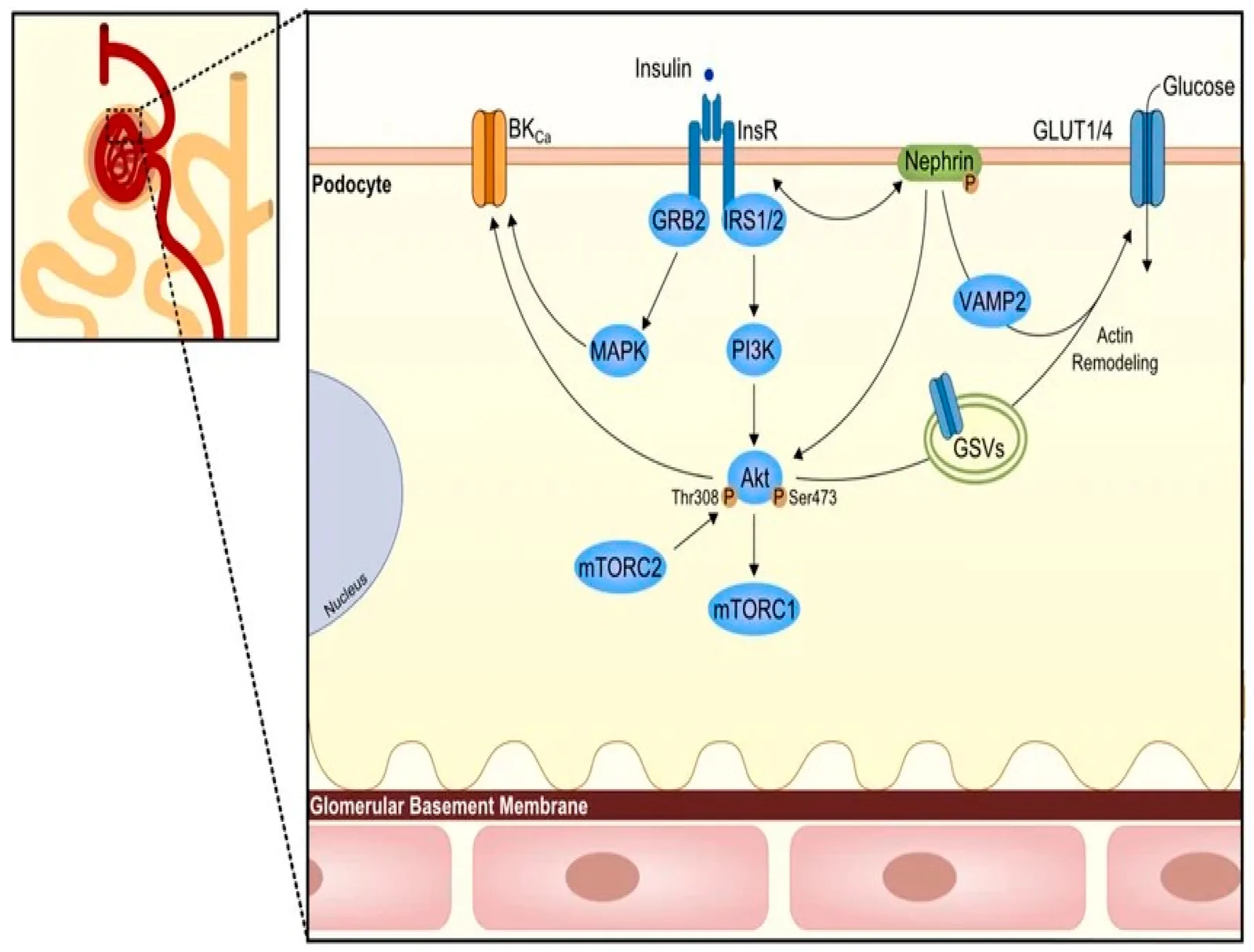
Insulin signaling in podocytes. Reprinted from ref. [51].

Podocytes are the first nephron cells that interact with insulin. After initiation via InsR/RTK, the signal is taken up by the same signaling pathways (Figure 2) [48,49]. Through these mechanisms, insulin exerts various effects, all vital for normal glomerular filtration. In these processes, an important role is played by the podocyte-specific protein nephrin. It forms the core structure of the glomerular slit diaphragm, acting as a molecular sieve to prevent proteinuria. It also acts as a signaling platform that regulates podocyte actin cytoskeleton activity, cell adhesion, polarity, and survival. Nephrin damage or its genetic mutation leads to severe renal disease [50]. It plays a role in the translocation of glucose transporters (GLUT1 or GLUT4) to the podocyte membrane and, consequently, promotes glucose uptake. The translocation appears to involve remodeling of Vamp2 (vesicle-associated membrane protein 2) and actin (phenomena initiated by the PI3K pathway). Other effects of insulin signaling on the PI3K and MAPK pathways in the podocytes are the maintenance of normal cytoskeletal (primarily actin) integrity and dynamics, and support for podocytes to adapt to pressure changes by increasing membrane expression and activity of BKCa channels (Large Conductance Calcium-Activated Potassium channels) and ensuring the promotion of cell survival through normal mitochondrial function and apoptosis inhibition [48,49].

Two multiprotein kinase complexes, mTORC1 and mTORC2 (mechanistic target of rapamycin kinase—mTOR), act as central regulators in these processes. mTORC1 promotes anabolic processes such as protein synthesis in podocytes, while mTORC2 regulates cell survival, metabolism, and cytoskeletal organization (Figure 2) [51].

High levels of insulin and glucose in T2DM, sustained over a prolonged period, cause insulin resistance and the disruption of these mechanisms, contributing to the development of diabetic nephropathy. Thus, excess insulin activates PKGIα (protein kinase GIα) and also induces oxidative stress through mitochondrial overactivation and production of ROS. PKGIα acts as a cysteine redox sensor that is directly activated by oxidation, forming interprotein disulfide bridges in cells exposed to ROS (H_2_O_2_), especially in podocytes [15,49]. Through these disulfide bridges, intracellular and membrane proteins modify their conformation and function. Insulin also stimulates the prolonged formation of BKCa membrane channels, which are activated by both extracellular and intracellular Ca^2+^. Under these conditions, a cytoskeletal reorganization occurs, through actin contraction, causing podocyte retraction and enlargement of the spaces between them, with the appearance of albuminuria [15,51].

**At the tubular level**, insulin binds to the same types of receptors as those on podocytes, intervening in glucose reabsorption, gluconeogenesis (inhibition) and natriuresis (inhibition).

*Glucose reabsorption.* Being a small molecule, glucose is completely filtered through the glomeruli and must be immediately reabsorbed in the proximal nephrocytes. Responsible for glucose reabsorption at the apical/luminal membrane are the sodium–glucose cotransporters SGLT2 (absorbs about 90% of filtered glucose) and SGLT1 (absorbs the remaining 10%) [52,53,54,55]. Renal glucose transport by SGLT2 is independent of direct insulin signaling. However, insulin exerts an indirect effect by stimulating the synthesis and membrane exposure of SGLT2. In diabetes, prolonged hyperglycemia is associated with increased membrane expression of SGLT2, because more glucose is excreted in the urine and must be reabsorbed [5]. On the other hand, antioxidants (such as N-acetylcysteine—NAC) block insulin from stimulating membrane expression of SGLT2 [56]. Recently, SGLT2 inhibitors, which lower blood glucose by blocking renal glucose reabsorption, independent of insulin, have become a therapeutic target in diabetes.

After absorption from the glomerular filtrate, glucose crosses the nephrocyte and is taken up by the GLUT2 transporter at the basolateral membrane (towards the peritubular capillaries) [52,53,54,55]. When blood glucose is normal, insulin stimulates GLUT2 to be positioned at this level [53]. In diabetes, hyperglycemia induces increased levels of filtered glucose. One of the observed changes was the movement of GLUT2 to the apical/luminal membrane and insertion at this level. Thus, in insulin resistance (type 2 diabetes), the kidney’s capacity to reabsorb glucose increases through the repositioning of GLUT2 in the luminal membrane. The result is the amplification of hyperglycemia [55,56].

*Gluconeogenesis.* One of the effects of insulin signaling through InsR is the inhibition of transcription factors, such as FoxO1. The result is a decrease in the synthesis of phosphoenolpyruvate carboxykinase (PEPCK) and glucose-6-phosphatase (G6Pase), key enzymes of gluconeogenesis [52]. Thus, liver gluconeogenesis inhibition occurs postprandially, when blood glucose is elevated, but renal gluconeogenesis paradoxically increases (often doubling) during this same period and is doubled by the increase in tubular glucose reabsorption. But during well-fed states, the kidneys switch priorities and readily consume glucose as an energy source rather than producing it [54,55]. During fasting periods, blood insulin levels drop greatly through the secretion of insulin antagonists, which activate gluconeogenesis. At the renal level, the phenomenon occurs predominantly in proximal nephrocytes, which use lactate and glutamine to produce glucose [15,54]. On the other hand, the glucose obtained is not consumed by nephrocytes (which use fatty acids as an energy source), but is delivered into the general circulation. It has been estimated that the kidney contributes 40% to overall gluconeogenesis, which is mainly carried out in the liver [54]. In increasing insulin resistance and in diabetes, the opposite situation occurs, in which renal gluconeogenesis is overamplified, worsening hyperglycemia [54].

*Natriuresis.* If glucose is reabsorbed in the proximal tubule, sodium is absorbed along the entire length of the tubules through various protein channels. Thus, approx. 65% of the filtered sodium is reabsorbed in the proximal tubule together with water, mainly through the Na-H exchanger type 3 (NHE-3) and SGLT proteins [57]. Then, another approx. 25% is reabsorbed in the ascending limb of the loop of Henle, through the sodium–potassium chloride cotransporter-2 (NKCC2). Finally, 5–10% of sodium is reabsorbed into the collecting duct through the epithelial sodium channel (ENaC). Only less than 10% of the filtered sodium is excreted in the urine [57]. Insulin stimulates sodium absorption in all the aforementioned tubular segments. In the proximal tubule, insulin regulates both the sodium transporters in the luminal membrane (NHE-3, SGLT2) and those in the basolateral membrane (Na/K-ATPase, NBCe1). For these actions, insulin stimulates an InsR2 receptor, while for gluconeogenesis it binds to InsR1 [58,59]. In a healthy individual, the physiological increase in insulin levels during postprandial periods induces a desensitization of IRS2, with a decrease in sodium reabsorption in the proximal tubule. The result will be an increase in its level in the distal convoluted tubule. When insulin resistance sets in, the desensitization mechanism is abolished and, therefore, sodium will not reach the distal tubule as much [15].

Insulin also indirectly influences sodium reabsorption, by interfering with the renin–angiotensin–aldosterone system. Thus, it increases plasma renin activity, with the consecutive production of greater amounts of angiotensin II and aldosterone [55]. The result is sodium and water retention, with the onset of hypertension. And through these indirect actions insulin counteracts the natriuretic effects of hyperglycemia.

Even though the kidney is not an insulin-dependent tissue, it actively participates in the insulin cycle in the body. Thus, two organs are involved in the normal process of recycling insulin: the liver, which metabolizes it, and the kidney, which metabolizes it and excretes it from the body. Being a small molecule, insulin is completely filtered through the glomeruli and is absorbed in very large proportions by the proximal nephrocyte, so that only 2% of the filtered insulin is lost in the urine [60]. In addition to glomerular filtration, it has also been proven that a significant amount of insulin is eliminated from the tubular capillaries (post-glomerulus) into the tubular cells, through the uptake of insulin by INSR from their basolateral membrane [60]. Whether it comes from the glomerular filtrate or from the peritubular blood, insulin is processed in the nephrocytes, through the following metabolic pathways:

- At the membrane level, it will be attacked by local proteases, a process that accounts for less than 2% of its degradation.
- In lysosomes: After they fuse with endocytosis vesicles, insulin is attacked by GIT (glutathione–insulin transhydrogenase), also known as insulin reductase or protein-disulfide reductase (glutathione). GIT is a specific enzyme that inactivates insulin by reducing the disulfide bonds that link the A and B chains. After the action of this enzyme, A and B chains are hydrolyzed by lysosomal proteases. This pathway is more active when higher than normal amounts of insulin end up in the urine [55,61].

It has been shown that the amount of insulin excreted in the urine is not influenced by the presence of glomerular pathology, but by that of tubular pathology. This confirms the role of the proximal nephrocyte in unlimited insulin reabsorption [55,60].

However, there are physiological variations in urinary insulin excretion (postprandial or fasting periods). Some pathological conditions should also be mentioned. Thus, in chronic kidney disease (CKD), insulin resistance has been observed in 30–50% of patients, even in the absence of diabetes. Also, increased insulin resistance induces a rapid and progressive alteration of CKD. Certain risk factors have been identified: sedentary lifestyle, disruption of adipokine secretion, metabolic acidosis, anemia of various causes, infectious foci generating inflammation and oxidative stress, and vitamin D3 deficiency [62].

It is assumed that the mechanisms involved are alterations in renal vascularization (through activation of the sympathetic autonomic nervous system), decreased Na^+^/K^+^ ATPase activity, sodium retention and increased glomerular filtration rate (GFR) [55,60].

Also, increased insulin resistance induces an increase in blood pressure through three associated mechanisms, which increase cardiovascular risk [60]:

- Increased activity of the renin–angiotensin–aldosterone system;
- Stimulation of the sympathetic nervous system;
- Decreased activity of the natriuretic peptide system.

**Despite all these data, the mechanisms by which insulin resistance induces CKD and the mechanisms by which CKD induces insulin resistance are not clarified.**

### 2.3. Actions of NT-3 Under Normal Conditions Versus Diabetes

**Scientific importance of the topic:** *The reader is informed about the functional particularities of NT-3 that differentiate it from other nerve growth factors (NGF and BDNF) and which are described at the level of signaling mechanisms. Also, the known actions exerted by NT-3 under normal conditions (both on nerve cells and on other cell types) will be systematized. Then, the actions of NT-3 in diabetic patients will be presented.*

We must begin with the reasons why NT-3 was selected as the subject of this review, among the entire neurotrophin family:

- Unlike NGF and BDNF, it can stimulate other types of cells than those of the nervous system, through a particular signaling mode, which will be presented below.
- Until the experimental study conducted by the authors of this analysis, NT-3 had never been theoretically approached and experimentally studied in patients with T2DM and ESRD and, most likely, not even in patients with ESRD alone.
- The results obtained and already published were interesting and sometimes very surprising, requiring further research. This review attempts to deepen the theoretical understanding of these results.
- For over 20 years, NT-3 has been the subject of the authors’ research in various pathologies (ischemic stroke, COPD, bacterial septic peritonitis) and has always demonstrated interesting behavior.

NT-3 belongs to the neurotrophin family, along with NGF (nerve growth factor), BDNF (brain-derived neurotrophic factor), and NT-4 (neurotrophin-4). All of these factors share common ancestral genes and structural similarities [5,63].

NT-3 was the third neurotrophic factor discovered, after NGF and BDNF, and is secreted in the central and peripheral nervous systems, heart, liver, and adipose tissue, but also in the pancreas and kidneys [6,64,65].

It is a 119-amino acid polypeptide homodimer with a molecular weight of 27.5 kDa. The two monomers are non-covalently linked. Each monomer contains a cystine knot motif formed by three disulfide bonds (positions 152–217, 195–246, and 205–248) that provide structural stability and enable binding to the TrkC receptor [66]. Its distribution and influence on different neuronal populations clearly differentiate NT-3 from NGF and BDNF. Classically, NT-3 is known to stimulate the genesis and survival of neurons in both the central and peripheral nervous systems. More recently, it has been shown that NT-3 also modulates synaptic transmission [63].

**Figure 3:**
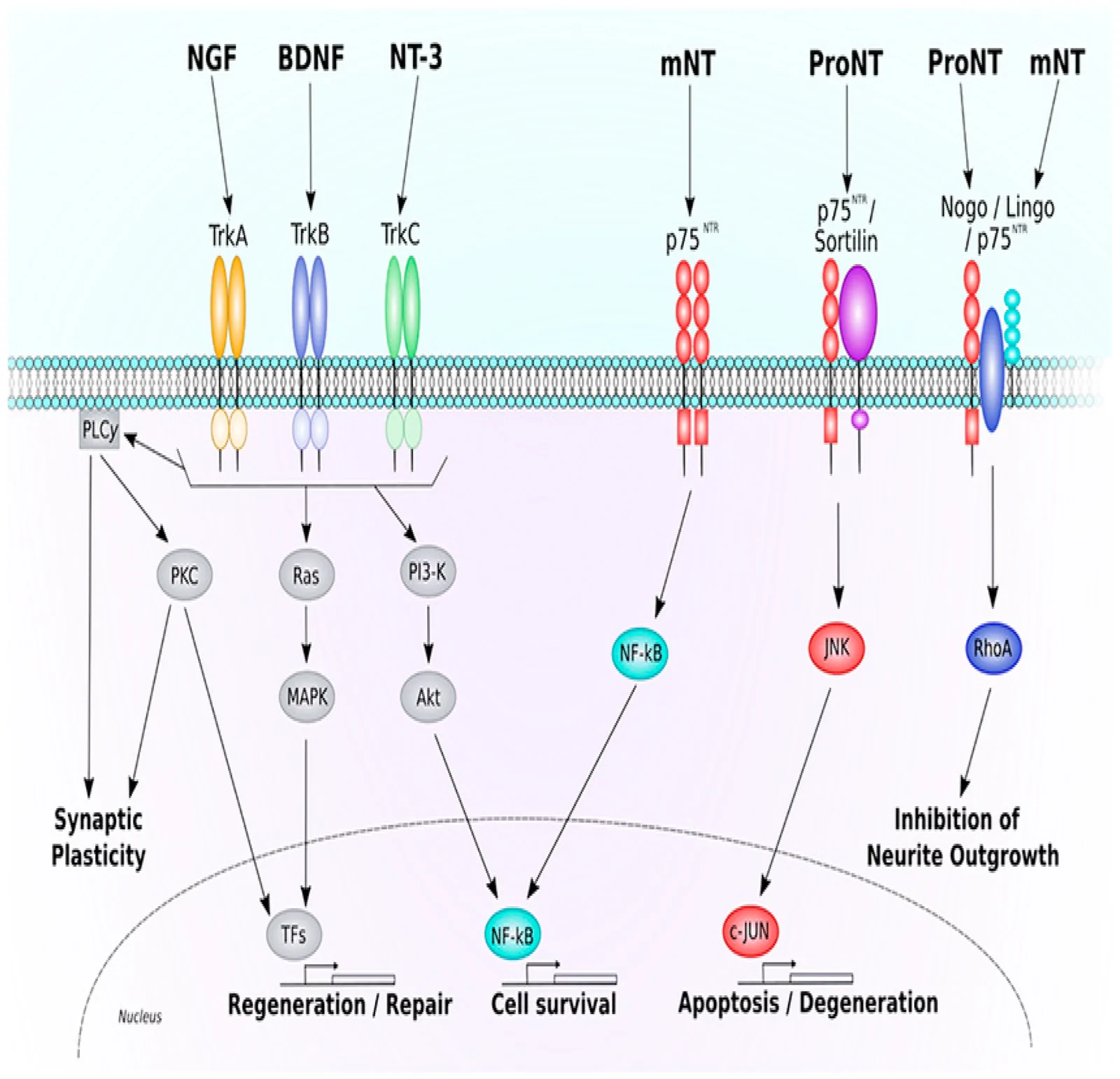
Preferential binding of nerve growth factors to membrane receptors and signaling pathways. It should be noted, however, that NT-3 can also bind to TrkA and TrkB, although it has the highest affinity for TrkC. mNT means mature neurotrophins, while pro-NT indicates the precursors of neurotrophins, which are also active molecules, with actions opposite to those of the mature forms. Reprinted from ref. [67].

**NT-3 is the only neurotrophin capable of activating all tyrosine kinase receptors used by neurotrophins (TrkA, TrkB, and TrkC).** It can also bind to the p75NTR receptor (belonging to the tumor necrosis receptor family), which is the first receptor discovered to be used by NT-3. NT-3 has a low affinity for this p75NTR that acts as a co-receptor (when co-expressed with Trk receptors, p75NTR acts as a critical co-receptor, forming a complex that significantly enhances the affinity and reactivity of Trk receptors to low levels of NT-3). **The highest binding affinity of NT-3 is for the TrkC receptor, which is only activated by NT-3 (**Figure 3**)** [5,63,66,67].

For receptor activation, NT-3 must dimerize, as determined by X-ray crystallography [68]. The dimeric state is very stable and crucial for signaling. By forming a dimer, NT-3 can bind simultaneously to two receptor molecules (TrkC and p75NTR), which are brought into proximity. Thus, a 2:2 ligand–receptor complex is formed. Unlike other neurotrophins, the binding affinity and specificity of NT-3 depend on the formation of a hydrogen bond between Gln 83 and Arg 103. It is known that Gln103 plays an important role in the specificity of binding, while Arg 103 is essential for the binding affinity to TrkC [68].

After high-affinity binding of NT-3 to TrkC, the receptors autophosphorylate and, in this form, recruit and activate PI3K. Activated PI3K phosphorylates phosphatidylinositol-4,5-bisphosphate (PIP2) to generate phosphatidylinositol-3,4,5-trisphosphate (PIP3) at the plasma membrane [68]. Accumulated PIP3 acts as a membrane anchoring site for proteins containing pleckstrin homology (PH) domains, most notably the serine/threonine kinase Akt (also known as protein kinase B) (Figure 3). The activated PI3K/Akt pathway promotes survival by inactivating pro-apoptotic proteins (like Bad-BCL2 associated agonist of cell death) and reducing apoptosis, making it a critical mechanism in neuronal survival against stressors [68].

In some contexts, especially at high concentrations, NT-3 can also activate TrkA or TrkB receptors, which can also trigger the PI3K/Akt as well as the Ras/MAPK (Mitogen-Activated Protein Kinase) and PLC-γ (phospholipase C gamma) pathways [68,69].

The results of these signaling pathways are the activation of the transcription factors CREB, NF-kB, ER81 (ETV1), Runx3, Olig1/2, and MEF2, with consecutive protein synthesis [68,69].

An attempt will be made to systematize the **known actions that NT-3 exerts in normal conditions.**

#### 2.3.1. Actions on Nerve Cells

a. NT-3 stimulates neuronal survival, differentiation, and regeneration. Therefore, NT-3 is present throughout the mature nervous system, but the highest concentrations are located in the hippocampus, cortex, and cerebellum. It plays a key role in neuronal survival and plasticity in these regions, especially in the dentate gyrus of the hippocampus [70].
b. NT-3 is also essential for the development and survival of subpopulations of sensory and motor neurons. It is highly expressed in areas containing proprioceptive neurons (spinal cord, muscles). Thus, large-diameter proprioceptive neurons in the dorsal root ganglia (which detect muscle stretch) are dependent on NT-3, in the absence of which they die [71].
c. Another proven aspect is the crucial role of NT-3 in the development, differentiation and long-term survival of auditory neurons (cochlear hair cells) and vestibular neurons, which are responsible for hearing and balance [72].
d. A very important function of NT-3 is to stimulate the development of the enteric nervous system, supporting the survival and differentiation of neurons in the intestinal myenteric and submucosal plexuses [73].
e. NT-3 is also involved in the development of sensory receptors in the skin (Merkel cells), muscle, and heart [74].
f. It also acts as a neurotrophic factor for sympathetic neurons in effector tissues such as the penis and bladder [75].
g. NT-3 regulates dendritic growth. Thus, in the developing cortex, it acts antagonistically to BDNF. More specifically, in certain layers, NT-3 inhibits BDNF-stimulated dendritic growth, while in others, it acts as its main stimulator.

Thus, NT-3 demonstrates a complex modulatory role, of high regional specificity, which differentiates it from NGF and BDNF, which have non-selective stimulatory actions [76].

#### 2.3.2. Actions Exerted on Other Cell Types

a. In addition to the actions presented above, NT-3 also has an **angiogenic effect**, both under normal conditions and in ischemic tissues (reparative neoangiogenesis), **a property not commonly attributed to NGF or BDNF** [68]. This role is exerted predominantly by the stimulation of TrkC receptors, followed by activation of the PI-3K-Akt-eNOS pathway [5,70,77]. Another known and important aspect is that NT-3 does not simultaneously participate with VEGFα in neovascularization [77]. As such, **a growth factor initially considered as only neuroregenerative in fact acts in a complex way, stimulating both neuronal and vascular regeneration.**
b. Another action of NT-3 is to stimulate the growth of some parenchymal cells. Thus, NT-3 secreted by hepatic stellate cells is discharged into the small space between them and hepatocytes. The result is the **activation of hepatocyte TrkB receptors, with cell proliferation** in the middle lobular regions [5,78].
c. NT-3 exerts **stimulatory actions on pancreatic cells**, both under normal conditions and in adenocarcinomas. Thus, it modulates cell survival, proliferation and differentiation, with a notable role in the progression of pancreatic cancer. Evidence shows the overexpression of NT-3 and its receptor TrkC in ductal adenocarcinoma [77,79]. **However, there is no evidence of direct stimulation of insulin secretion by NT-3.**
d. Increased levels of NT-3 and TrkC receptors are also proven to be found **at the renal level,** without their actions being clearly known [5,80]. These aspects will be detailed in Section 3.
e. NT-3 exhibits distinct immunomodulatory actions: it decreases microglial activation and reduces macrophage infiltration into damaged tissues, also having an inhibitory effect on the pro-inflammatory cytokines IL-1β and TNF-α. These **anti-inflammatory actions** have not been identified for NGF [81].

#### 2.3.3. NT-3 Actions in Diabetic Patients

Before starting the presentation of experimental data from the literature, it is useful to recall the actions of NT-3 on the endocrine pancreas. The data are not numerous. In insulin-secreting INS-1 cells, NT-3 induces rapid phosphorylation of the TrkC receptor, without increasing insulin secretion; it only increases intracellular calcium levels.

On the other hand, NT-3 stimulation of TrkC receptors on the membranes of pancreatic α cells induces glucagon synthesis. This means that **NT-3’s actions at the cellular level are enhanced by an improvement in energy metabolism, secondary to the entry of glucose into the cells. And glucose enters efficiently when there is hyperglycemia.**

**It is very important to remember, though, that glucose enters cells efficiently in hyperglycemia only in insulin-independent tissues.** Which are these tissues? The brain (neurons and astrocytes), but not peripheral neurons, the blood–brain barrier, erythrocytes, the liver (hepatocyte glucose uptake for storage or release is generally independent of insulin, although insulin suppresses gluconeogenesis), the kidney (medulla), and the intestinal mucosa. All of these tissues use the glucose transporter GLUT1. On the other hand, insulin-dependent tissues such as skeletal muscle and adipose tissue mainly use GLUT4, which requires insulin to move to the cell surface [82].

a. Studies in diabetic patients have shown increased levels of NT-3 in skin biopsies, reflecting damage to local nerve fibers due to neuropathy [83]. NT-3 has also been investigated in the retina of diabetic patients, showing that it stimulates angiogenesis and maintains vascular integrity [84].
b. Another important aspect experimentally highlighted was the significant increase in serum NT-3 levels in patients with diabetic neuropathy after physical exercises for the diabetic foot [85]. It is interesting to note that skeletal muscle cells achieve insulin-independent glucose uptake during exercise. Although resting muscle cells are insulin-dependent, during contraction an alternative, insulin-independent mechanism is triggered that allows glucose uptake even when insulin levels are low or when insulin resistance is present [85].
c. A study on nerve growth factors in diabetic patients used muscle biopsies taken from the gastrocnemius and deltoid muscles. The expressions of NGF, BDNF, NT-3, NT-4 and ciliary neurotrophic factor (CNTF) were determined for these samples. The results obtained showed that NT-3 decreased in striated muscles of diabetic patients, being associated with muscle weakness and manifestation of neuropathy. This study proves the important pro-regenerative effects of NT-3 at the level of the nerve endings that innervate skeletal muscles [86].

### 2.4. Aspects Highlighted by the Authors in Their Previous Studies Regarding NT-3 in Hemodialysis Patients with Type 2 Diabetes Mellitus

**Scientific importance of the topic:** *The reader is informed about the serum behavior of NT-3 in the two categories of patients studied. The results were extremely polarized. As this is the first and only study conducted to date on this topic in patients with both T2DM and ESRD, functional interpretations have to be based exclusively on the data from this study.*

**The purpose of this subchapter of the manuscript is not to make a detailed presentation of these results, which have already been published. But there are no other results in the specialized literature regarding the behavior of NT-3 in patients with ESRD that associate it with T2DM. Furthermore, the results obtained were quite surprising.**

To enable the reader to better understand the depth of the study, its general aspects will be briefly outlined.

The study conducted in 2024 targeted two categories of patients: the test group was represented by patients with T2DM and ESRD, and the control group was made up of patients with ESRD only (Table 1) [5,6,7]. Although the study did not include very many patients, the sample size used was absolutely representative of any dialysis patient in a clinic in Bucharest (2 million inhabitants) with the same equipment and performance as those in the study.

**General research plan**

The patient groups included in the study were investigated according to three separate research directions:

- The genetic study of the intestinal microbiota, in order to identify possible microbial alterations and their impact on the immunological behavior and underlying pathology of the patients;
- The complex study of innate immunity (inflammation) and acquired immunity (cellular immunity);
- The study of tissue regeneration capacity.

**Methods**

- **The genetic study of the gut microbiota:** special techniques that are not based on cultivation were used, since most of the species in the intestinal flora are not cultivable [7].

Bacterial DNA was extracted from stool samples using a commercial kit (Qiagen Stool Mini Kit; Qiagen, Inc., Germantown, MD, USA). The DNA concentrations were quantified utilizing a Qubit 4 fluorometer (Thermo Fisher Scientific, Inc., Waltham, MA, USA). In order to investigate the gut microbiota, 16S ribosomal RNA (rRNA) and 18S rRNA primers were used and quantitative PCR was performed [7].

The following bacterial populations were investigated: *Actinobacteria, Deferribacteres, Verrucomicrobia, Tenericutes, Betaproteobacteria, Epsilon proteobacteria, Gamma proteobacteria Mucispirrilum, Gammatimonadetes, Eubacteria, Lactobacillus, BPP, Clostridium leptum, Clostridium cocoides, Ruminococcus, Firmicutes, Bacteroidetes sp., F. Prausnitzii, ARNr 18S, Saccharomyces* and *Candida* [7].

- **The immunological study** had two targets: innate immunity (inflammation) and adaptive immunity (this was chosen to address the cellular immune response because, unlike the humoral one, it is insufficiently studied in this category of associated pathologies). This part of the study was essential because, although the influence that each of the two pathologies exerts on the body’s immune response is known, their combined effect is much less studied [5,6,7].

As markers for innate immunity (inflammation), the following were determined: IL-6 (interleukin 6), sIL-6R (soluble IL-6 receptor), IL-1β (interleukin 1β), TNFα (tumor necrosis factor α), IL-10 (interleukin 10), and NGAL (neutrophil gelatinase-associated lipocalin). On the other hand, TNFβ (tumor necrosis factor β)/LTα (Lymphotoxin α) was determined as a marker for adaptive immunity (cellular immune response) [5,6,7].

- **The study of tissue regeneration capacity** was performed by measuring the serum levels of neurotrophin-3 (NT-3) and vascular endothelial growth factor β (VEGFβ). Although it was mentioned that this is the only study found in the literature that investigates NT-3 in patients with T2DM and ESRD, it should also be said that VEGFβ is another marker very rarely studied in the two associated pathologies. To find correlations with the period of post-inflammatory tissue regeneration, the behavior of growth factors was compared with the serum level of the anti-inflammatory cytokine Il-10 [5,6,7].

All the aforementioned compounds were determined from serum samples, utilizing Merck Millipore ELISA kits for IL-6, IL-1β, IL-10, NT-3, and VEGF β, and Elabscience ELISA kits for sIL-6R, TNFα, TNFβ, and NGAL.

**Statistical Analysis**

For the comparison between groups, the data were processed using the SPSS IBM V26 statistical package, with statistical significance defined as *p* ≤ 0.05, conventionally adopted for checking the degree of data compatibility with the null hypothesis. The statistical protocol was applied according to the design of a cross-sectional study and the type of data collected. Descriptive statistics, such as mean ± standard deviation, median, and range, were used for quantitative variable presentation (such as age, duration of hemodialysis, and serum markers). For comparison between groups (T2DM patients vs. NON-DM patients, IL-6, sIL-6R, etc.), an independent-samples *t* test or nonparametric tests (independent-samples Mann–Whitney U test, Kruskal–Wallis test) were applied after data normality distribution was verified. Qualitative variables (such as gender, and presence or absence of T2DM) were described as structure or intensity indicators (absolute or relative frequencies). The chi square (X^2^) or Fisher’s exact test was also applied to examine the association between the categorial variables. Correlation analysis was also performed. Boxplot graphs (of the minimum, first quartile, median, third quartile, maximum, and outliers of the dataset’s parameters) were created to show the data series distribution for the global sample or stratified in subgroups of interest [5,6,7].

**Results**

The results obtained are systematized in Table 2.

**Figure 4:**
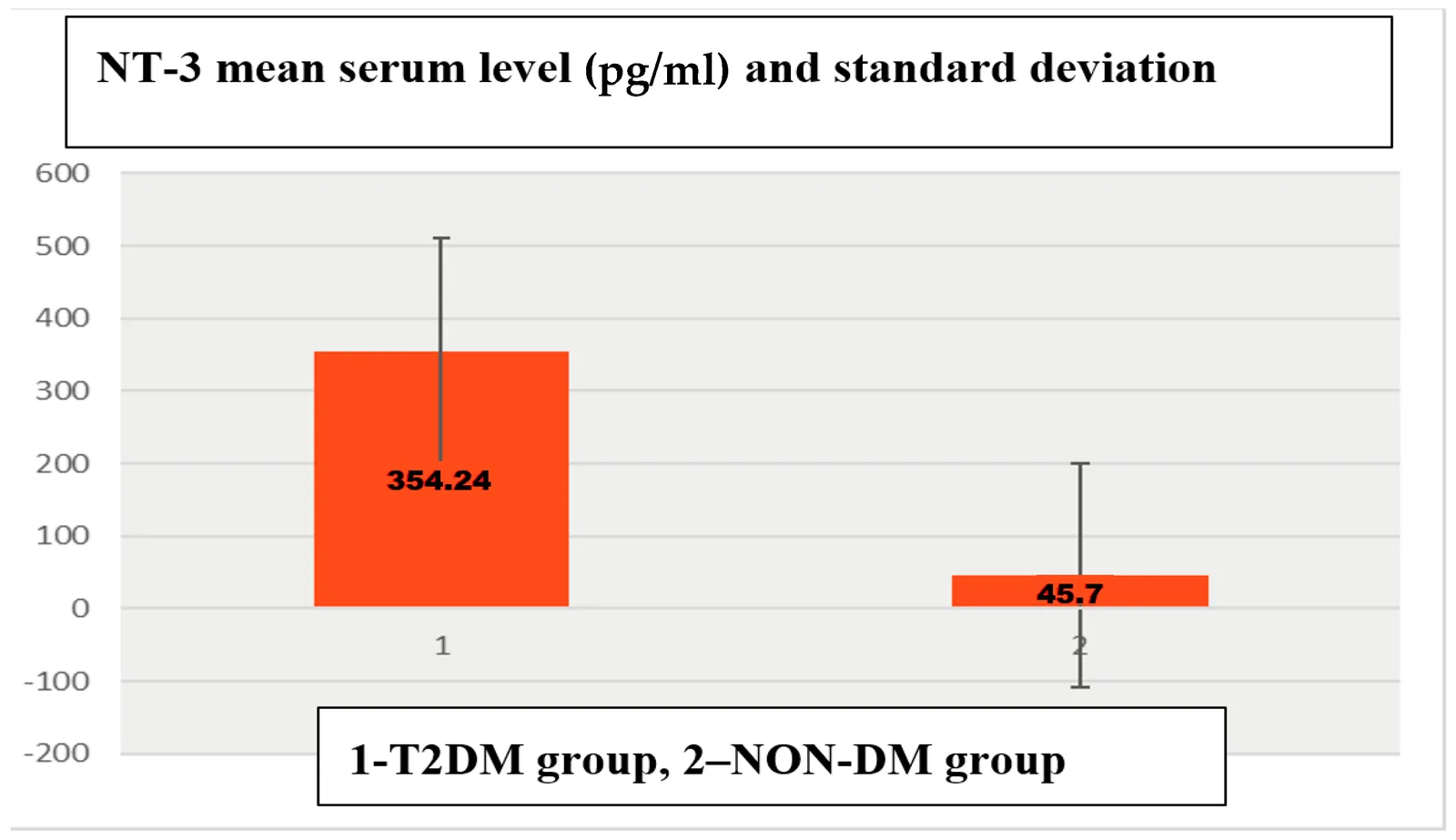
The inhomogeneous behavior of NT-3 in both studied groups (the standard deviations were very large) [5,6,7].

For the test group (patients with T2DM and ESRD/T2DM), an average serum NT-3 level was obtained that was almost 8 times higher than in the control group (only ESRD/NON-DM), with the difference being statistically significant (354.24 ± 916.64 pg/mL vs. 45.7 ± 254.64 pg/mL) (Figure 4). The difference was statistically significant at *p* = 0.02 [5,6,7].

Thus, the serum level of NT-3 in hemodialysis patients with T2DM is 7.8 times higher than in NON-DM patients. The maximum serum level of NT-3 in the test group was 3594.43 pg/mL, compared to 1718.71 pg/mL in the control group (2 times higher) (Table 2) [5,6,7]. This maximum value contributed the most to generating a very large standard deviation and belonged to a special case that has already been published in the scientific literature [6].

The values obtained in the two groups were compared with the upper limit of the internationally accepted normal range. The mean serum NT-3 level was more than 15 times higher in the test group and more than 2 times higher in the control group [5,6].

The first highly unexpected aspect was the presence of very high maximum values: in the test group, the maximum NT-3 was almost 180 times higher than the international upper limit, while in the control group it was almost 86 times higher. We can deduce that the maximum value in the test group was double that in the control group.

Because we initially thought there were dosing errors, we repeated the quantitative dosages, but the results obtained were identical.

**Figure 5:**
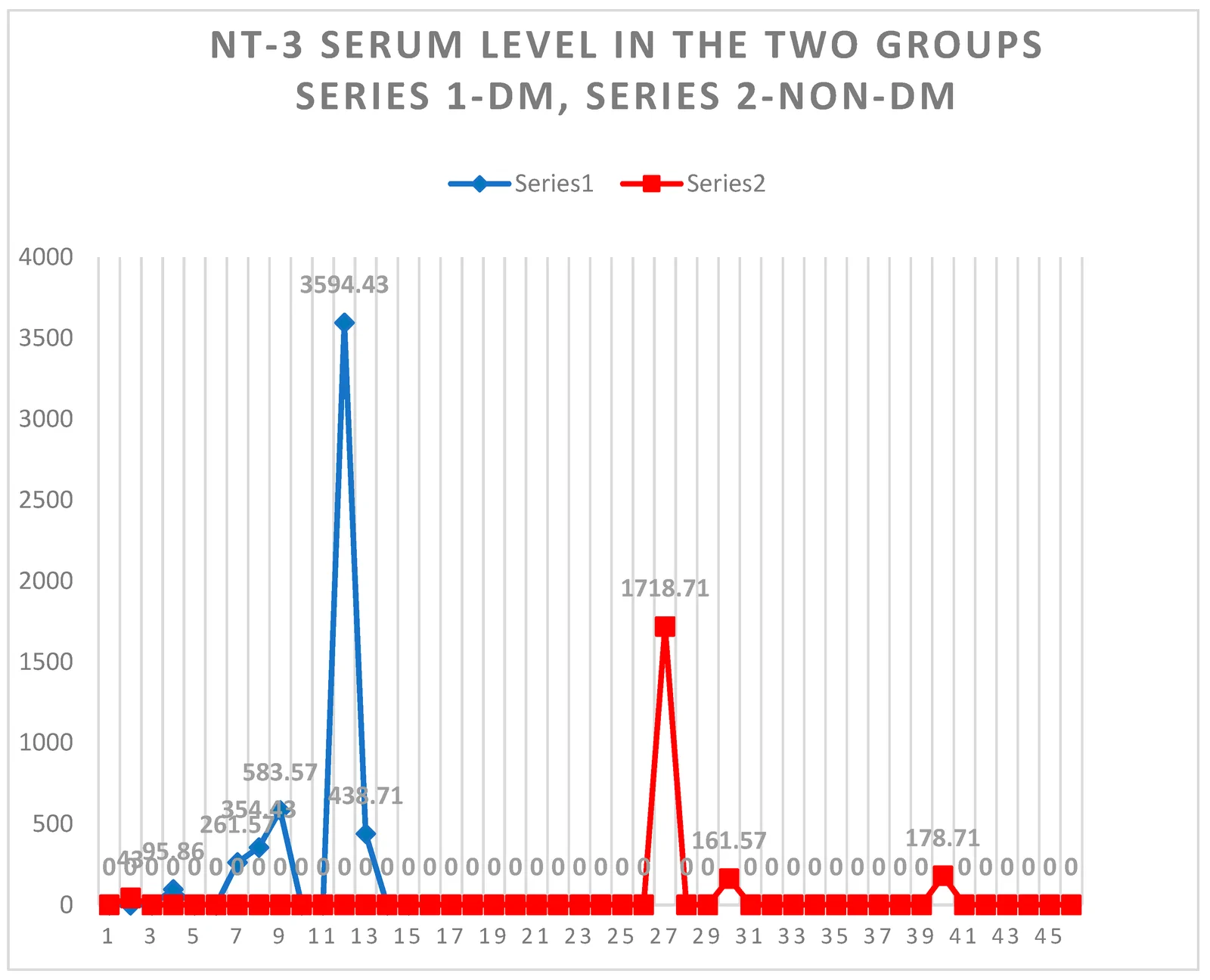
A few patients in both groups had non-zero NT-3 values, some of which were highly elevated [5,6,7].

But what surprised us the most was the **extremely inhomogeneous behavior of NT-3 in both groups** of patients studied (Figure 4). Thus, the standard deviations were very high. More specifically, only 40% of the patients in the test group had a serum level of NT-3 above the detection limit of the kit used, while in the control group only 8% achieved this (Table 2) (Figure 5) [5,6,7].

**From the beginning, we noticed a dual behavior in patients with the same diagnosis: some of them showed very high serum concentrations of NT-3, while most showed an inhibited synthesis capacity for this growth factor. What could be the explanation?**

As already mentioned, elevated levels of TrkC receptors for NT-3 have been shown to be present in the kidney, although the consequences of their activation are not clearly known [80]. What is known for sure is that NT-3 stimulates the development and survival of neuronal precursors in the human embryonic kidney and inhibits bicarbonate reabsorption in the ascending limb of the loop of Henle in the adult mouse kidney [5,87,88].

In addition to nervous system cells, both podocytes and tubular nephrocytes present TrkC receptors. Thus, the actions of NT-3 at the renal level are certain [86]. However, the number of TrkC receptors on glomerular podocytes is higher than on tubular nephrocytes. This indicates the important role of NT-3 for proper glomerular function [5,6,89]. On the other hand, the role of NT-3 at the tubular level has not been clarified [89].

Combining these data, it can be concluded that **NT-3 actually controls proper glomerular function through the podocytes that coat the glomerular capillary wall** [5,6].

However, another very interesting aspect should be mentioned: **glomerular podocytes are insulin-dependent cells, with their metabolic, structural and survival functions also being maintained by insulin signaling. Although glomerular podocytes are highly sensitive to insulin, adjacent glomerular endothelial cells are unresponsive to insulin** [90].

It was observed that **podocytes are stimulated by both NT-3 and insulin. A possible explanation is that, to respond optimally to NT-3 stimulation, podocytes require insulin to take up glucose and boost their energy metabolism.**

However, there is also a fundamental difference between the two podocyte activators: their very different serum levels (as we mentioned in the first part of this paper, insulin has a serum level approximately 100 times higher than that of NT-3) [8,9]. It can be stated that the actions of NT-3 on podocytes are of functional importance, even though, compared to insulin, NT-3 originates from a lower quantitative level of the fractal reality represented by the human body.

Why is the term **“fractal reality”** used?

It is known that in physics and mathematics, a fractal is a complex pattern that exhibits self-similarity across different scales—meaning the same structural motifs repeat themselves from the macro-scale down to the microscopic level. But very recent and very well-argued scientific studies validate the fractal structure and functioning of the human body [91].

**But what happens in ESRD?**

Histologically, the characteristic changes consist of diffuse glomerulosclerosis and vascular sclerosis, as well as severe tubulointerstitial fibrosis, leading to tubular atrophy. Additional features include tubular obstruction by protein casts, which causes tubular dilation, and chronic inflammation [5,6,92]. **The result is the disappearance or drastic reduction of TrkC receptors on the podocyte membrane. Consequently, NT-3 has extremely limited or no opportunity to exert its effects.** These histological changes have long been documented in patients from both study groups, allowing us to infer the functional consequences as well.

The cells responsible for these histological transformations are fibroblasts. Chronic inflammation and local mechanical stress generate massive production of TGFβ (transforming growth factor beta), which stimulates resident fibroblasts for a long time. The result is transdifferentiation into myofibroblasts, which express alpha-smooth muscle cell-specific actin (α-SMA) and produce excessive collagen, with fibrosis of the renal tissue and loss of its functions. The genetic mechanism underlying this transdifferentiation is the activation of the Spp1 gene in fibroblasts, following signaling through the TGFβ/Smad pathway [93].

It is important to note that **both fibroblasts and myofibroblasts are insulin-sensitive cells, without being insulin-dependent. In diabetes, the insulin signaling pathway in fibroblasts is altered by hyperinsulinemia or hyperglycemia, which induces fibrosis and excessive differentiation of fibroblasts into myofibroblasts** [94]. **On the other hand, there is no direct evidence indicating that renal fibroblasts or myofibroblasts express TrkC (so NT-3 cannot act on these cells).**

Returning to our research, we must try to find explanations for the two opposing trends that the studied groups presented.

**a. Why did the majority of patients studied (60% of those in the test group and 92% of those in the control group) exhibit very low serum NT-3 values, below the detection limit of the kit used?**

This behavior could have the following explanations [5,6]:

- Very low general capacity for neuroregeneration, neuronal survival and functional control of synapses, even though these are essential in diabetic and uremic neuropathy.
- Angiogenic capacity reduction or inhibition via NT-3 antagonists, even in the presence of diabetic arteriopathy. This is merely a theoretically possible explanation, since, at present, no endogenously produced NT-3 antagonists are known, only synthetic TrkC receptor blockers.
- Cancellation of renal NT-3 production, both in podocytes and in tubular cells.
- Although the patients selected for the two groups showed no clinical or paraclinical signs of acute inflammation, the majority exhibited elevated to very high serum IL-6 levels, a finding that confirms the presence of an intense chronic inflammatory state in the patients within the studied groups [7]. It is already proven that, in high concentrations, IL-6 inhibits BDNF synthesis [95]. This aspect was addressed in another study by the authors and already published: chronic inflammation with high serum levels of IL-6 also inhibits NT-3 secretion [7].

**b. Why did 40% of the patients in the test group and 8% of those in the control group show highly elevated serum NT-3 values? What could be the explanation for this extremely polarized behavior?**

Due to the severe renal histological changes in ESRD, amplified by T2DM, it can be hypothesized that the increased serum NT-3 levels do not originate from the kidneys. As mentioned above, fibroblasts and myofibroblasts become the majority cells of the renal parenchyma in ESRD. Although these cells are known to secrete NT-3 in the skin and heart [96], there is no evidence of this secretion in the kidney. As mentioned before, they also do not express TrKC for NT-3.

Most probably, the sources of NT-3 are ischemic tissues and chronic inflammatory foci (caused by uremia and dialysis), rather than the kidneys. It must be reiterated that none of the patients included in the study exhibited acute ischemic and/or inflammatory syndrome at the time of blood sample collection [5,6].

In addition, it is important to remember that the patients with the maximum NT-3 values in the two groups are elderly and very close in age (82 and 83 years, respectively), so it is very likely that NT-3 is secreted via degenerative nervous and cardiovascular tissues changes [6].

It can be concluded that this polarization of serum NT-3 values in the two groups involves the following aspects:

- As a result of histological changes and their functional consequences, ESRD contributes to a decrease in serum NT-3 levels; this is primarily explained by a marked reduction in its renal synthesis within nerve endings, podocytes, and tubular cells, structures that are all significantly reduced in number. It is worth noting that this phenomenon occurs even in the presence of uremic neuropathy. Although there are no studies quantifying the level of renal NT-3 production, the aforementioned statement is plausible, given that NT-3 synthesis in the normal kidney is a well-established fact.
- Even though ESRD is associated with T2DM, **diabetic neuropathy and angiopathy could counteract the negative renal influence on serum NT-3 levels in only 40% of the patients in the tested group. In the other 60%, the counteracting phenomenon is absent or insignificant.**
- The very high serum levels observed in a small subset of patients from both groups indicate extrarenal production of NT-3, most likely originating from degenerated nervous and cardiovascular structures. At first glance, this extrarenal production appears to be chronic. However, it is possible that **these extremely high NT-3 levels precede the onset of acute ischemia in various parts of the body, thereby announcing severe acute clinical events.** We propose this hypothesis because the 82-year-old female patient in the study group—who had exhibited peak serum values for several of the analyzed markers, including NT-3—died a few months after the study concluded. Her relatives could not be contacted, and the dialysis center staff were unaware of the exact cause of death. However, upon interviewing all the fellow dialysis patients who knew her, we were told they had learned that the cause of death was a stroke [5].

## 3. Discussion

**Scientific importance of the topic:** *The reader is invited to examine the bidirectional functional connections between insulin and NT-3, as inferred by the authors through a comparison of two sources of information: the specialized literature and the results obtained from the direct study. This direct study is the only one conducted on NT-3 in patients with T2DM and ESRD; consequently, no other reference data exist. Furthermore, no other published information regarding the functional connections between insulin and NT-3 in this patient population was found.*

**Possible functional connection between insulin and NT-3 in hemodialysis patients, especially in those with T2DM**

To present these little-known and rarely addressed functional links in the aforementioned patient categories, it is relevant to examine the two logical directions of biological information exchange:

a. The influence of insulin on NT-3 synthesis and actions at the renal level;
b. The influence of NT-3 on insulin synthesis and actions at the renal level.

We will revisit some aspects presented in the first part of this paper, but everything will be presented in a different light, adding new information.

**a.**  **Insulin’s influences on renal NT-3 synthesis and actions in patients with ESRD and T2DM**

According to the specialized literature, there are no data indicating that insulin directly stimulates the synthesis of NT-3. Furthermore, there are no data on the functional cooperation between insulin and NT-3 in these two categories of patients. It can only be hypothesized that, being a protein anabolic, insulin generically stimulates protein synthesis, including that of NT-3, but there are no experimental results indicating the direct action of insulin to increase NT-3 expression.

**However, what is known with certainty is that podocytes and renal tubular cells, structures that synthesize NT-3, are sensitive to insulin [90].**

Of the two types of kidney cells that secrete NT-3, glomerular podocytes are the more sensitive to insulin [90]. As was specified in the first part of this paper, they also express the glucose transporters GLUT1 (which is not insulin-dependent, but responds to insulin stimulation) and GLUT4 (which is insulin-dependent). It was shown how insulin stimulation of podocytes is crucial for maintaining the actin cytoskeleton, cellular morphology, and the integrity of the glomerular filtration barrier [90]. In T2DM, insulin signaling in podocytes is altered or even abolished, with the appearance of cytoskeletal and membrane lesions, proteinuria and the onset of diabetic nephropathy [90,97]. In fact, prolonged exposure of human podocytes to insulin induces insulin resistance through lysosomal and proteasomal degradation of the insulin receptors [96]. Secondary to this degradation, signaling through the PI3K/MAPK pathways used by insulin is greatly diminished, so impaired glucose absorption will appear [97]. Through this mechanism, podocytes become insulin-resistant and will have a lower capacity to produce ATP.

Reintroduction of the InsR into the podocyte membrane can re-trigger the signaling cascade, but cannot fully restore glucose uptake. **This highlights the very complex structural and functional connections of insulin receptors, and the fact that not only their numerical decrease induces insulin resistance** [97].

**How do these changes in insulin receptor signaling influence NT-3 synthesis and actions in podocytes?**

It is evident that the energy deficit induced by reduced glucose uptake in podocytes impairs their protein anabolism. We can infer that the podocytes’ capacity to synthesize NT-3 (a protein) and membrane receptors (glycoproteins) is also diminished.

On the other hand, **NT-3 signaling through TrkC uses the same PI3K as insulin, with the difference that, after PI3K, Akt kinase is activated and not MAPK (as in insulin signaling)** (Figure 1) [67]. At first glance, it might be considered that insulin deficiency enables an increase in the availability of PI3K kinase in podocytes for NT-3 signaling. But, as has been said, the protein synthesis deficit targets the synthesis of both NT-3 and TrkC receptors, as well as intracellular proteins, including Akt kinase. This same protein synthesis deficit also affects signaling via TrkA and TrkB receptors, to which NT-3 might bind with low affinity.

It can be logically inferred that the mechanisms described are even more pronounced in ESRD, particularly when associated with T2DM, since it is known that the number of active podocytes is significantly reduced in this condition.

**What happens in tubular nephrocytes?**

As was also mentioned in the first part of this manuscript, renal tubular cells, especially proximal ones, have a very high number of insulin receptors, which regulate essential functions through their signaling: glucose reabsorption (through sodium–glucose cotransporter 2—SGLT2), albumin absorption and sodium transport [98].

Insulin resistance in these cells appears through the same mechanism as in podocytes and leads to reduced receptor expression (through decreased protein synthesis) and mitochondrial dysfunction, with the triggering of prolonged oxidative stress and consequent structural damage. These tubular mechanisms also contribute to the development of diabetic nephropathy [99].

It is known that under physiological conditions, insulin receptors on proximal tubule cells suppress or inhibit renal gluconeogenesis. Targeted deletion of insulin receptors in proximal nephrocytes, or the impairment or blockade of insulin signaling via these receptors (as seen in insulin resistance), abolishes this inhibitory control. The result is the stimulation of gluconeogenesis, a process that, in turn, contributes to elevated glucose levels both locally and systemically [100].

On the other hand, under normal conditions, NT-3 is synthesized and expressed in renal tubular cells, particularly in distal nephrocytes, where it performs functions other than those traditionally associated with the nervous system. It has been shown that NT-3 regulates various transport mechanisms and functions of tubular nephrocytes via TrkC receptors and cAMP- and PKA-mediated signaling [88]. Specifically, NT-3 acts primarily on the medullary thick ascending limb (MTAL) of the loop of Henle, inhibiting bicarbonate reabsorption [87].

**What effects can diabetic nephropathy and ESRD have on local and systemic NT-3 synthesis?**

Based on all the data presented, we can state that in diabetic nephropathy and ESRD (whether or not associated with T2DM), there is a decrease in both the number of NT-3-producing cells (podocytes and tubular cells) and the number of TrkC receptors on their membranes. However, in the absence of other experimental data, we must rely solely on the findings of our study regarding NT-3 behavior in patients with T2DM and ESRD.

On the other hand, our study did not perform intrarenal quantification of NT-3, but only serum levels. It is important to, once again, note that there are no other data in the literature quantifying the physiological or pathological renal production of NT-3. Our data indicated a significant increase in the mean NT-3 level of the test group, compared to the non-diabetic control group (nearly eightfold higher), leading us to consider a possible role for NT-3 as a marker of severe metabolic alterations.

However, the extreme polarization of patient behavior should not be overlooked: although the majority of subjects in both groups exhibit extremely low NT-3 levels, many falling below the detection limit of the kit used, there are a few with high values, which resulted in the dramatic increases observed in the means and standard deviations.

However, given that all these patients demonstrably have extremely low numbers of nephrons and their constituent cellular elements, is it logical to claim that these markedly elevated serum NT-3 levels originate in the kidneys? These arguments support the conclusion that in none of these patients did the elevated serum NT-3 originate from the kidneys.

The enhancement of gluconeogenesis in the pathological kidney was discussed earlier, and it was emphasized that this process leads to an increase in local and systemic glucose levels. Another question arises that requires experimental verification: does this hyperglycemic phenomenon perhaps promote NT-3 signaling within insulin-independent structures?

By increasing gluconeogenesis, does the kidney with T2DM and ESRD not indirectly stimulate the extrarenal actions of NT-3, even though it can no longer be produced in the kidney?

**b.**  **Potential influences of NT-3 on insulin synthesis and actions at the renal level**

It has been shown that NT-3 modulates glucose metabolism, counteracting high blood glucose levels and insulin resistance associated with diabetic neuropathy and obesity. One way to achieve these effects is to stimulate glucose consumption in peripheral tissues for energy production [101].

Even though NT-3 exerts autocrine and paracrine stimulatory actions on pancreatic cells, modulating their survival, proliferation and differentiation, there is no evidence to prove direct stimulation of insulin secretion by NT-3. Although it has been shown to trigger signaling through the TrkC receptor in mouse pancreatic beta cells, the observed result was an increase in intracellular calcium concentration and not in insulin directly [102]. This phenomenon may represent a key step in both the initiation of insulin secretion and the apoptosis of these cells.

Although NT-3 does not exert direct action to stimulate or inhibit pancreatic insulin secretion, it interferes both directly and indirectly with the actions of insulin in podocytes and renal tubular cells. This interference is explained by the fact that insulin and NT-3 use the same kinase (Akt) to initiate signal transduction in these renal cells, even though the receptors are different.

In addition, **activation of podocyte TrkC receptors by NT-3 also results in activation of insulin-like growth factor-1 receptor (IGF-1R); thus, NT-3 participates in modulating insulin signaling** [89]. Insulin and the insulin-like growth factors (IGF-1 and IGF-2) are closely related peptides that use the same signaling pathway (PI3K/Akt).

**Details:** It is known that TrkC signaling (and thus NT-3 signaling) is not essential for nephron development but is important for maintaining glomerular integrity during aging. Specifically, mice with a TrkC deficiency (and consequently a lack of NT-3 signaling) in the nephron exhibit signs of glomerular senescence: enlarged glomeruli accompanied by mesangial proliferation, basement membrane thickening, effacement of podocyte foot processes (accompanied by albuminuria), podocyte loss, and features characteristic of focal segmental glomerulosclerosis (FSGS) [89]. Thus, normal TrkC expression (and, consequently, normal NT-3 signaling) is essential for glomerular health.

On the other hand, it has been shown that NT-3-mediated activation of podocyte TrkC receptors induces signaling via insulin-associated ErbB and TLR pathways [89]. More specifically, NT-3-mediated activation of TrkC on the podocyte membrane triggers IGF-1R activation through phosphorylation of tyrosine residues [89]. Another important finding is the identification of increased TrkC expression in the glomerular tissue of patients with diabetic nephropathy [89].

In conclusion, NT-3 signaling via TrkC is essential both for maintaining glomerular health and for the transactivation of IGF-1R in podocyte cultures (in vitro) and in mouse kidneys (in vivo).

Thus, an interconnected signaling network is formed (the IGFs-INS axis, doubled by the NT-3 pathway) that regulates cell growth, metabolism, and survival. Although insulin mainly regulates glucose metabolism, and IGFs, like NT-3, regulate cell growth and differentiation, these molecules share structural aspects, receptors, and signaling pathways through which they can interact [103].

**Conclusions:**

**1.**  **There is unequivocal experimental evidence demonstrating that the stimulation of TrkC receptors by NT-3 is essential for renal cell survival.**
**2.**  **The information presented above constitutes arguments suggesting that NT-3 stimulates insulin action in the kidney under normal conditions.**

**But what happens in diabetes and ESRD?**

It has already been shown that the mechanisms of diabetic nephropathy and the progressive onset of end-stage renal disease (ESRD) lead to a reduction in the number of podocytes and renal tubular cells (both being sources of NT-3), as well as a decrease in the number of TrkC receptors on their membranes.

After highlighting the interferences between insulin, IGF1 and NT-3 signaling, it becomes clear that **the decrease in NT-3 synthesis and TrkC signaling could induce insulin and IGF1 signaling deficits.**

Another phenomenon that should not be overlooked is the fact that, as CKD progresses, **a decreasing number of functional renal cells are stimulated by NT-3 produced either by hypoxic intrarenal nerve endings (secondary to diabetic angiopathy) or by systemic neurons under conditions of diabetic neuropathy.**

**It is well known that excessive growth factor stimulation of a cell affected by a metabolic disease (diabetes, in the case of the patients discussed) triggers apoptosis in that cell** [104]. **Thus, not only is the stimulation of renal cell survival reduced, but the risk of the remaining cells undergoing apoptosis also increases.**

This statement is based on two arguments: the kidney simultaneously experiences not only a decrease in local NT-3 production, but also an increase in systemic NT-3 levels. In addition to apoptosis of residual renal cells, this aspect could also predict severe acute nervous and cardiovascular suffering (as we have information that this occurred with the patient who presented the maximum NT-3 value).

**Another important phenomenon involves fibroblasts, myofibroblasts, and mesangial cells.** They do not secrete NT-3. But all these cell types are insulin-sensitive, without being insulin-dependent. Hyperinsulinemia and/or hyperglycemia present in diabetes amplify insulin signaling in fibroblasts, myofibroblasts, and mesangial cells, which become energetically overactivated. These cells are also highly functionally interconnected. The mesangial cell is very similar to myofibroblasts. It has cytoplasmic extensions/processes that extend from the cell center towards the capillary lumen. Within these extensions are fibrils with a diameter of 7–10 nm, through which the mesangial cell is connected to the surrounding matrix and the glomerular basement membrane, with respect to the endothelial cells [105,106]. **Along with fibrosis induced by hypersecretion of collagen from fibroblasts and myofibroblasts, hyperactivated mesangial cells play an important role in the development of diabetic nephropathy and the onset of ESRD by secreting matrix proteins that deposit in the center of the glomerulus [107].**

## 4. Conclusions

- **Under normal conditions, there are strong arguments for the existence of indirect mutual stimulation between insulin and NT-3, at both the renal and systemic levels.**
- **Insulin and NT-3/TrkC signaling interact at the renal cellular (podocyte) level.**
- **On the other hand, it is very probable that in T2DM and ESRD this interaction develops into a vicious circle that accentuates the progressive loss of renal cells, most likely through apoptosis.**
- **The only cells that “benefit” from the intrarenal metabolic environment are fibroblasts, myofibroblasts, and mesangial cells. These cells become hyperactivated and, in addition to fibrosis induced by collagen hypersecretion, play a significant role in the development of diabetic nephropathy and the onset of ESRD through the secretion of matrix proteins that deposit in the glomerular center.**
- **As this represents the first and only study on this topic involving this category of patients, the proposed functional connections between insulin and NT-3 require clinical validation as well as further experimental and prospective research.**

## 5. Proposals for New Therapeutic Possibilities and Future Directions for Patient Monitoring

The therapeutic effect of NT-3 on neuronal structure and function has already been proven. Thus, NT-3 administration restores axonal caliber, prevents the decrease in sensory nerve conduction velocity, and stops the proximal accumulation of proteins in the neurofilaments of sensory neurons [108,109].

The angiogenic potential of NT-3 has also been exploited therapeutically. This potential is proven in ischemic limbs and tissues, contributing to their repair (which is very useful in treating diabetic foot ulcers) [110,111].

Recently, new cyclic mimetic molecules of BDNF and NT-3 have been discovered, which activate trophic signaling pathways and protect elderly brains from neurodegeneration [112].

**Could these protective and regenerative properties of NT-3 not be therapeutically exploited at the diabetic kidney level to prevent/slow down the onset of ESRD?**

**Based on the arguments presented in this paper, could these properties become therapeutic targets in podocytes and tubular nephrocytes as well (and not just in renal nerve endings and vascular walls)?**

## 6. Directions for Further Research

This study on the serum behavior of NT-3 in hemodialysis patients with type 2 diabetes mellitus represents only an initial step in this line of research and involved a relatively small group of subjects. It needs to be expanded. However, it allowed the opening of new research directions.

- Does periodic administration of NT-3 to diabetic patients, in doses that need to be experimentally established, reverse/slow the progression to chronic kidney disease and ESRD?
- Could the extremely high serum levels of NT-3 present in some of the patients studied also make it possible to stimulate the other two types of receptors, TrkA and TrkB, for which NT-3 has a low affinity? It is known that high affinity of a ligand for its receptor means activation of the receptor by small amounts of ligand, while low affinity has the opposite meaning.

What is the difference between NT-3’s actions mediated through TrkC receptors and those mediated by NT-3 through TrkA and TrkB?

What are the renal specificities of these actions?

- Although insulin has a serum level approximately 100 times higher than NT-3, they act similarly at the cellular level (on tyrosine kinase receptors) and potentiate each other. Does this mean that, deep in our structures, there is no qualitative difference between signaling through a molecule with endocrine action (and a single tissue source) and another with predominantly auto- and paracrine action (with multiple tissue sources)?
- Although NT-3 acts predominantly in an auto- and paracrine manner, it also passes into the blood, exerting actions at a distance from the tissue of origin. Does NT-3 “carry” the biological information of the tissue from which it originates at a distance?

Can the NT-3 molecule constitute a means of informational exchange between different tissues?

## Figures and Tables

**Table 1:** General information about patients included in the study [7].

	Average Age	Gender M/F	CKD Duration	Hemodialysis Duration
ESRD/non-T2DM (45)	61.5 ± 15.1 years	33/12	1–24 years	5.29 ± 4.71 years
ESRD with T2DM (15)	63 ± 11.8 years	9/6	1–14 years	2.53 ± 2.69 years

**Table 2:** Serum behavior of NT-3 in the two groups of patients studied. Abbreviations: C_mean_—average serum level, ULN—upper limit of the internationally accepted normal serum level.

Parameters Related to NT-3	NT-3 C_mean_ (pg/mL)	C_mean_/ULN of NT-3 RATIO	Maximum NT-3 Serum Level	Maximum/ULN NT-3 Ratio	Serum Level of NT-3 Above the Detection Limit of the Kit Used
**Test group/control group** **Ratio**	**8**	**15/2**	**2**	**2/1**	**5/1**
**Test group** **(T2DM + ESRD)**	**354.24**	**15**	**3594.43**	**180**	**40% of the patients**
**Control group** **(ESRD)**	**45.7**	**2**	**1718.71**	**86**	**8% of the patients**

## Data Availability

No new data were created or analyzed in this study. Data sharing is not applicable to this article.
